# Learning Curve Analyses for Left Bundle Branch Area Pacing with Conventional Stylet-Driven Pacing Leads

**DOI:** 10.1155/2023/3632257

**Published:** 2023-05-18

**Authors:** Ga-In Yu, Tae-Hoon Kim, Hee Tae Yu, Boyoung Joung, Hui-Nam Pak, Moon-Hyoung Lee

**Affiliations:** ^1^Division of Cardiology, Department of Internal Medicine, Severance Hospital, Yonsei University College of Medicine, Seoul, Republic of Korea; ^2^Division of Cardiology, Department of Internal Medicine, GyeongSang National University Changwon Hospital, Gyeongsang National University College of Medicine, Changwon, Republic of Korea

## Abstract

**Background:**

Physiological conduction system pacing has attracted attention to overcome the dyssynchrony problems of conventional right ventricular pacing (RVP). Left bundle branch area pacing (LBBAP), which complements short combing of His bundle pacing (HBP), has emerged and has proven its efficiency and safety. In addition, initial experiences of LBBAP were mainly using lumen-less pacing lead, and the feasibility of stylet-driven pacing lead (SDL) was also established. The purpose of this study is to evaluate the learning curve for LBBAP using SDL.

**Methods:**

The study enrolled 265 patients who underwent LBBAP or RVP performed by operators without previous LBBAP experience at Yonsei University Severance Hospital in Korea between December 2020 and October 2021. LBBAP was performed using SDL with an extendable helix. The learning curve was evaluated by analyzing fluoroscopy and procedure times. And, before and after reaching the learning curve, we evaluated how much the time required for the LBBAP differed from the time required for the RVP.

**Results:**

LBBAP was successful in 50 of 50 (100.0%) patients left bundle branch pacing was successful in 49 of 50 (98.0%). In 50 patients who underwent LBBAP, mean fluoroscopy and procedural times were 15.1 ± 13.5 minutes and 59.9 ± 24.8 minutes, respectively. The plateau of fluoroscopy time reached in the 25th case and the plateau of procedure time reached in the 24th case.

**Conclusion:**

During the initial experience with LBBAP, fluoroscopy and procedural times improved with increasing operator experience. For operators who were experienced in cardiac pacemaker implantation, the steepest part of the learning curve was over the first 24-25 cases. It is shorter than the previously reported learning curves of HBP.

## 1. Introduction

Conventional right ventricular pacing (RVP) can cause electrical and mechanical dyssynchrony and is associated with increased risks of cardiac remodeling, pacemaker-induced cardiomyopathy, heart failure, and mortality [1, 2]. These deleterious effects have driven the search for alternative sites for physiological pacing. His bundle pacing (HBP), developed as the early conduction system pacing (CSP), showed the result of preserving synchronous ventricular activation by directly activating the His–Purkinje conduction system [3, 4]. However, HBP has limitations such as the difficulty of the procedure because his area is very small and the need for high pacing output [5, 6].

Left bundle branch area pacing (LBBAP), proposed by Huang et al. in 2017 [7], has emerged as a new physiological conduction system modality that has a lower and more stable threshold and achieves a paced QRS duration similar to that of HBP [8]. In addition, long-term efficiency and safety have been proven through several studies [9, 10]. Moreover, initial experiences of LBBAP were mainly with lumen-less pacing lead (LLL) [7, 11]. And recently, the feasibility and safety of LBBAP using standard stylet-driven pacing lead (SDL) was also established [12–14].

LBBAP presents a simpler implantation technique, shorter fluoroscopy duration, and a procedure time with a steeper learning curve compared to HBP. However, most of the learning curve analyzes of LBBAP reported so far have been procedures using LLL [10, 15]. In addition, there is a lack of research on when one operator can stably perform the LBBAP procedure.

The purpose of this study is to evaluate the learning curve of one operator of LBBAP using SDL. We confirmed that the procedure was performed stably through the lead and electrocardiogram (ECG) parameters, success rate, and complication rate of the procedure. And, by analyzing fluoroscopy and procedure time, we reported a learning curve for how many cases were needed per operator to get used to the procedure. Furthermore, differences in required radiation dose and procedure time when compared with existing RVP before and after achieving the learning curve of LBBAP were evaluated.

## 2. Methods

### 2.1. Study Population

**Figure 1:**
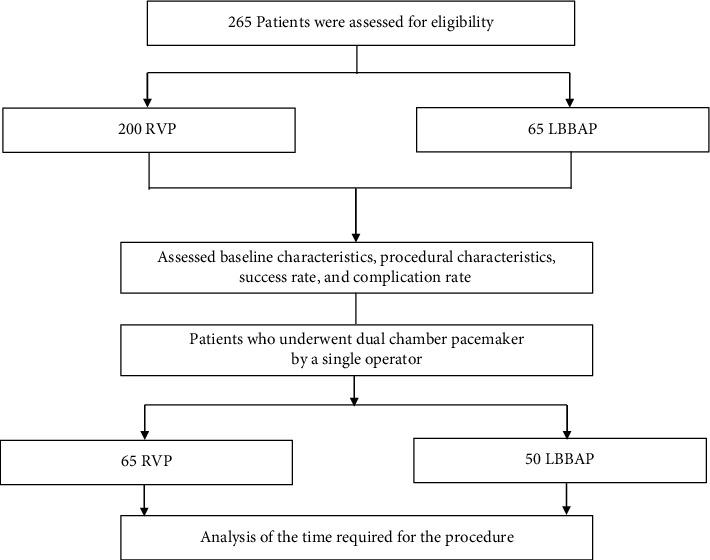
Flowchart of the enrolment for analysis: right ventricular pacing (RVP) and left bundle branch area pacing (LBBAP).

This retrospective observational study enrolled patients who underwent conventional RVP (*n* = 200) or LBBAP (*n* = 65) performed by operators without previous LBBAP experience between December 2020 and October 2021 at Yonsei University Severance Hospital in the Republic of Korea. All patients who required ventricular lead implantation during this period were included. For the learning curve analysis, patients who underwent dual-chamber pacemaker implantation by a single experienced operator were divided into a group that underwent conventional RVP (*n* = 65) and a group that underwent LBBAP (*n* = 50) (Figure 1). This study was performed following the ethics of the Declaration of Helsinki (2013) of the World Medical Association and approved by the Institutional Review Board of Yonsei University Health System (4-2023-0053). Following strict confidentiality guidelines, personal identifiable information was removed after database creation, and therefore, the study was exempt from prior consent requirement.

### 2.2. Procedural Technique for LBBAP

All procedures, including conventional RVP and LBBAP, were performed by operators without previous LBBAP experience. LBBAP was performed with a 5.6-Fr sized SDL with an extendable helix (Solia S60, Biotronik SE & Co., Berlin, Germany) and all the SDL delivered through a preshaped sheath (Selectra 3D, Biotronik SE & Co., Berlin, Germany) because it properly targets the pacing site and maintains sufficient backup during lead implantation.

The lead was prepared as previously described by De Pooter et al. [13]. Briefly, it was prepared by exposing the extendable screw by turning the outer pin 10 to 12 times clockwise, followed by five additional clockwise turns of the outer pin using the standard stylet guide tool delivered with the lead to avoid partial unwinding of the extendable helix. The initial choice of sheath was to select a midlength and midsize curve (Selectra 3D-55-39) that was more suitable for the size of the heart. Consequently, the sheath used changes to a smaller (Selectra 3D-40-39) or larger (Selectra 3D-65-39) curve depending on the size of the patient's heart.

Two methods were used to find the optimal site for LBBAP. Initially, tagging the His area using His/right ventricular (RV) catheter, then set that point as a landmark, place the lead tip at 1-2 cm toward the RV apex in the right anterior oblique (RAO) view and perpendicular to the septum in the left anterior oblique view as described in previous studies (Supplementary Figure S1A) [7, 16]. When his potential could not be confirmed with the His/RV catheter, the nine-partition fluoroscopic method was used. In this method, a RAO fluoroscopic image of the ventricle is divided into nine sections and two specific partitions (high and median septum middle areas) as LBB areas, and the leads were placed by targeting these areas (Supplementary Figure S1B) [17, 18].

In the site described previously, we advance the pacing lead by fast rotation 5–10 times with keep the stylet in pacing lead until the final position is reached. 12 lead surface ECGs and intracardiac electrograms were continuously monitored using an electrophysiology recording system. Left bundle branch (LBB) capture was confirmed using published criteria. With the advancement of the lead, QRS configuration with a right bundle branch block (RBBB) observed in surface *V*1 ECG, suggesting the site of pacing at the LBB. At this point, during pacing the right side of the interventricular septum, a fast peak left ventricular (LV) activation time in surface *V*5-*V*6 ECG of approximately 75–85 ms by programmed stimulation should be noted, which demonstrates the transition from deep ventricular septal pacing to LBBAP (Supplementary Figure S2A). When the pacing lead is near or at the LBB, an LBB potential can be recorded (Supplementary Figure S2B) [7, 10, 18, 19]. To avoid complications such as ventricular septal perforation, the lead impedance was continuously monitored during the screw, and advance was stopped immediately if there was a sudden drop in lead impedance [14, 20]. After confirming the LBB capture, the sheath was removed.

### 2.3. Definition of RVP and LBBAP

RVP was defined as conventional RV apical pacing. LBBAP included deep septal pacing (DSP) and left bundle branch pacing (LBBP). The LBB potentials from the pacing lead were recorded and stimulus to left ventricular activation time was abruptly shortened (Stim-LVAT) which were confirmed as LBB capture. In details, If the RBBB configuration was seen in *V*1 during the unipolar pacing in addition to one or more of the following findings, the success of LBBP was confirmed: (1) LBB potentials is recorded from the pacing lead, with the potential to ventricle interval of 15–35 ms [21–23]; (2) Stim-LVAT in *V*5-*V*6 is shortens abruptly and measured <75–85 ms. Stim-LVAT is defined as the interval from the pacing stimulus to the peak of the R-wave and is often used to reflect the lateral precordial myocardium depolarization time in leads *V*5-*V*6 [11, 18, 22]; (3) QRS morphology transition reflecting LBB pacing during the threshold test [20, 22]; and (4) QRS morphology change by the programmed stimulation from pacing lead [22]. When LBB capture was confirmed, it was defined as LBBP [21–23]. Even in the absence of LBB capture, confirmed it to be a DSP if it provided more synchronous LV activation, defined as a pacing QRS of 125 ms compared to RVP [24].

### 2.4. Statistical Analysis

Descriptive statistics were used to organize and interpret patients' baseline characteristics and comorbidities. Categorical variables are reported as frequencies (percentages), and continuous variables are reported as mean ± standard deviation or median (interquartile range). Categorical variables were compared using Fisher's exact test or Pearson's chi-square test, whereas continuous variables were compared using Student's *t*-test and Wilcoxon's rank-sum test. A paired *t*-test was performed to compare the measured values before and after the procedure. The fluoroscopy and procedure times were modeled as cubic spline functions. The cubic spine curve was mathematically differentiated and the point where the slope gradient converged to zero and forms a plateau was analyzed (Supplementary Figure S3). A linear regression analysis was performed to confirm whether the decrease in fluoroscopy and procedural time, followed by an increase in experience. All tests were two-tailed with values of *p* < 0.05 considered as significant. Statistical analyses were performed using R programming version 4.0.3 (The *R* Foundation for Statistical Computing, Vienna, Austria).

## 3. Results

### 3.1. Baseline Characteristics

A total of 200 patients who underwent conventional RVP (mean age 70.47 ± 12.01 years, 49.5% female) and 50 patients who underwent LBBAP (mean age 68.75 ± 14.94 years, 50.8% female) were included in this study.

The distribution of pacing indications was as follows: in the conventional RVP group, sick sinus syndrome (SSS) was the most common indication (54.0%), followed by atrioventricular (AV) block (46.0%): in the LBBAP group, AV block was more frequent (81.5%) than SSS (18.5%). The QRS duration was more likely to be narrow in the conventional RVP group (62.0%) than in the LBBAP group (40.0%). A comparison of baseline characteristics among the groups is summarized in Table 1.

### 3.2. Electrophysiological Characteristics of LBBAP

Among 65 patients who underwent LBBAP, 60% of patients had baseline QRS widening (*n* = 39), and among them, RBBB, LBBB, and bifascicular block accounted for the highest frequency with the same number of 13.8% (*n* = 9). There was no statistical difference in overall baseline QRS morphology between the SSS and AV block groups (*p*=0.30). All patients who underwent LBBAP showed reduced QRS duration; the duration decreased from 128.6 ± 30.2 ms at baseline to 120.5 ± 20.1 ms after LBBAP. When the SSS was the pacing indication, the decrease in QRS duration was 7.50 (−17.66 to 32.66, *p*=0.52) ms, and when the AV block was the pacing indication, the decrease in QRS duration was 8.18 (−0.03 to 16.41, *p*=0.05) ms. Intraprocedural electrophysiological characteristics of LBBAP are summarized in Table 2.

### 3.3. Learning Curve of LBBAP Using SDL

**Figure 2:**
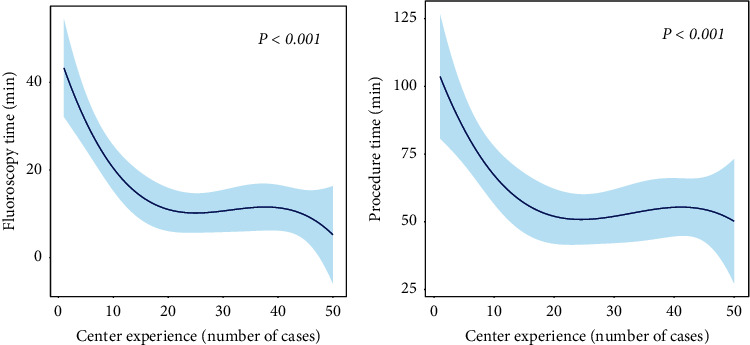
Time required for LBBAP according to center experience. Nonlinear cubic spline curves shown for the fluoroscopy time (a) and the procedure time (b) according to center experience. Blue line represents the fitted line of the association between number of cases and time required for LBBAP according to center experience, whereas the shaded region represents the 95% confidence interval; LBBAP, left bundle branch area pacing.

To eliminate bias, analyses were performed on patients who underwent the same procedure performed by a single operator. In 65 patients who underwent conventional RVP, the mean fluoroscopy and procedural time were 5.6 ± 3.2 and 42.7 ± 15.5 minutes, respectively. Of the 50 patients who underwent LBBAP, the mean fluoroscopy and procedural times across all procedures were 15.1 ± 13.5 and 59.9 ± 24.8 minutes, respectively. The fluoroscopy and procedure times for LBBAP were modeled as cubic spline functions, and the curve showed that both fluoroscopy and procedure time decreased with operator experience (Figure 2).

To confirm the plateau phase of the time required for LBBAP, the cubic spline curve was differentiated with respect to the procedure time to find the point where the slope was zero. In this analysis, the plateau phase of the procedure time for LBBAP began in the 24th case (Supplementary Figure S3).

**Figure 3:**
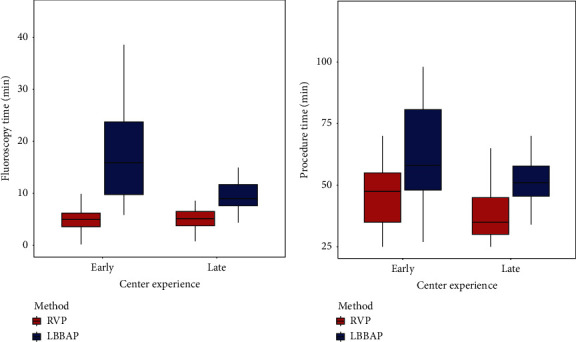
Median time required for LBBAP before vs. after reaching the plateau phase. Before and after achieving learning curve for the LBBAP, the fluoroscopy (a) and the procedure time (b) required for RVP vs. LBBAP is shown. LBBAP, left bundle branch area pacing; RVP, right ventricular pacing.

We evaluated how much effort required for the LBBAP procedure was different from that required for the conventional RVP after reaching the learning curve for LBBAP. Based on the 24th case whose procedure time reached the plateau phase, the entire study period was divided into early period and late period (before plateau phase ≤24th case vs. after plateau phase >24th case). Then, the difference between the procedure time for the LBBAP and the procedure time for the conventional RVP at the two periods were analyzed. In the early period, the mean procedure time of RVP and LBBAP were 46.7 ± 11.5 and 68.1 ± 32.4 min, respectively, and the difference was 21.4 (7.05 to 35.78, *p*=0.004) min. In the late period, the mean procedure time of RVP and LBBAP were 40.4 ± 17.1 and 52.4 ± 10.9 min, respectively, and the difference was 12.0 (5.19 to 18.84, *p* < 0.001) min. In the early period, the mean fluoroscopy times of RVP and LBBAP was 5.6 ± 3.4 and 20.5 ± 17.7 min, respectively, and the difference was 14.9 (7.39 to 22.54, *p* < 0.001) min. In the late period, the mean fluoroscopy times of RVP and LBBAP were 5.6 ± 3.0 and 10.0 ± 3.6 min, respectively, and the difference were 4.4 (2.71 to 6.11, *p* < 0.001) min. The time required for RVP and LBBAP before and after achieving the learning curve for LBBAP was presented as Figure 3.

### 3.4. Procedural Outcomes of LBBAP

LBBAP, which includes LBBP and DSP, was successful in 50 of 50 patients (100.0%), and LBBP, meaning LBB capture, was successful in all 50 to 49 patients (98.0%). There was 1 case of LBBP fail in the early period. After the procedure, the LBBAP lead parameters were sensed *R* wave amplitude of 10.8 ± 4.8 mV, ventricular capture threshold of 1.0 ± 1.2 V (at 0.4 ms), and ventricular pacing impedance of 675.7 ± 97.5 Ω. Baseline QRS duration was 127.7 ± 29.6 ms and paced QRS duration was 117.2 ± 19.0 ms (Table 3).

There were two cases of complications during the early period, which is LBBAP lead dislodgements occurring 1 day and 3 months after the procedure, respectively. Of the two dislodgement of ventricular lead cases, one patient required ventricular lead revision and LBBAP was achieved again, and one case did not require revision because of stable parameters with a ventricular lead in the RV inferior wall. There were 2 cases of pocket hematoma in the late period, and no procedure-related major complications occurred during that period (Table 3).

## 4. Discussion

The major findings of this study were as follows: (1) the LBB capture threshold and pacing impedance in the LBBAP population were stable, comparable to values in general RVP; (2) after LBBAP, QRS duration was significantly decreased; (3) time required for LBBAP procedures continued to decrease as increasing operator experience and achieved a plateau starting from the 24th case.; and (4) higher success rates and lower complication rates were achieved as experience increased.

Although HBP, first introduced by Deshmukh et al. in 2000, has been suggested as the ideal approach for physiological ventricular activation [25, 26], wider clinical application of HBP is limited by several problems, including technical difficulties in identifying the precise location, variable success rates, and potential risk of premature battery depletion and lead revisions due to progressive increases in capture thresholds [26, 27]. In this regard, LBBAP, which has a stable and lower capture threshold and a similarly paced QRS duration to HBP, has emerged as a new physiological conduction system modality and long-term efficiency and safety have been proven [8–10, 28]. Moreover, initial experiences of LBBAP were mainly with LLL, however more recently, the feasibility and safety of LBBAP using conventional SDL was also established [12–14]. In symptomatic bradycardia requiring frequent right ventricular pacing, CSP which improves synchrony is recommended and LBBAP, which is easier to access than HBP, may be considered more. The possibility of LBBAP using SDL, which was used for conventional RVP, is expected to expand the LBBAP.

In this study, we evaluated the learning curve of LBBAP using SDL, the acute outcome of the procedure before and after achieving the learning curve, and the difference in time for procedure from the time required for existing RVP. The plateau phase of the procedure time for LBBAP using SDL began from the 24th case, which is shorter than the previously reported learning curves of HBP (30–50 cases) even LBBAP using LLL [15, 29, 30]. Throughout the entire study period, the procedure time of LBBAP was significantly higher than that of RVP; however, the mean difference decreased to 21.4 min in the early period and 12.0 min in the late period.

There was a significant difference in the procedure time before and after achieving the learning curve; however, there was no statistically significant difference in the outcome of the procedure such as acute success rate, pacing parameter, and paced QRS duration in this study. These results make it possible to expect a stable procedure outcome even if an operator who has no experience of performs the LBBAP using SDL. This is encouraging considering the shortcoming of technical difficulties in identifying the precise location, variable success rates, and potential risk of premature battery depletion and lead revisions due to progressive increases in capture thresholds of HBP, the first CSP [26, 27].

Procedure-related major complications occurred in the early period in all 2 out of 2 total cases. On the other hand, ventricular septal perforation, a known complication of LBBAP, did not occur during the entire duration of this study. This is expected in accordance with our method to prevent complications. We continuously monitored the lead impedance during screwing to avoid ventricular septal perforation and advance was stopped immediately if there was a sudden drop in lead impedance. In the early period, if the lead impedance showed a rapid drop even if it was 500 ohms or more, we did not advance further and fixed the lead in that position. Referring to previous studies [18, 20] and accumulated our experience, although we maintained lead impedance monitoring, but when the lead impedance dropped below 500 ohms, it was regarded as a sign of septal perforation, so there was a more active advance than in the early period, lead stability may have increased. This careful lead impedance monitoring would have been the basis for avoiding ventricular septal perforation.

### 4.1. Study Limitations

This study has several limitations. First, this was a retrospective, observational, and nonrandomized study. Second, we purposely analyzed the experience of a single operator with the aim of reducing bias, but we believe that the results are broadly applicable. Comparing the experiences of multiple physicians in multiple centers would have better ensured its generalizability.

## 5. Conclusions

During the initial experience of LBBAP using SDL, procedural and fluoroscopy times continued to improve with operator experience. For physicians experienced in cardiovascular implantable electronic device implantation and without previous LBBAP experience, the steepest part of the learning curve was over the first 20–25 cases.

## Figures and Tables

**Table 1:** Baseline characteristics of patients.

	RVP (*N* = 200)	LBBAP (*N* = 65)	*P* value
*Demographics*			
Age (years)	70.4 ± 12.0	68.7 ± 14.9	0.886
Female sex	99 (49.5%)	33 (50.8%)	0.972
Body mass index	23.9 ± 3.4	24.1 ± 3.6	0.752
*Pacing indications*			
Sick sinus syndrome	108 (54.0%)	12 (18.5%)	<0.001
AV block	92 (46.0%)	53 (81.5%)	<0.001
Second degree AV block	5 (2.5%)	5 (7.6%)	
High degree AV block	19 (9.5%)	12 (18.5%)	
Complete AV block	33 (16.5%)	34 (52.3%)	
Others^*∗*^	35 (17.5%)	2 (3.1%)	
Baseline QRS morphology			<0.001
Narrow QRS	124 (62.0%)	26 (40.0%)	
RBBB	33 (16.5%)	9 (13.8%)	
LBBB	31 (15.5%)	9 (13.8%)	
Bifascicular block	0 (0.0%)	9 (13.8%)	
Trifascicular block	0 (0.0%)	5 (7.7%)	
Paced rhythm	12 (6.0%)	7 (10.8%)	

Values are presented as mean ± standard deviation or *n* (%). ^*∗*^Consist of chronotropic incompetence, atrial standstill with junctional bradycardia and heart failure for cardiac resynchronization therapy device. RVP, right ventricular pacing; LBBAP, left bundle branch area pacing; AV block, atrioventricular block; RBBB, right bundle branch block; LBBB, left bundle branch block.

**Table 2:** Electrophysiological characteristics of LBBAP.

	All (*N* = 65)	SSS (*N* = 12)	AV block (*N* = 53)	*P* value
Baseline QRS morphology				0.305
Narrow QRS	26 (40.0%)	6 (50.0%)	20 (37.7%)	
RBBB	9 (13.8%)	0 (0.0%)	9 (17.0%)	
LBBB	9 (13.8%)	3 (25.0%)	6 (11.3%)	
Bifascicular block	9 (13.8%)	1 (8.3%)	8 (15.1%)	
Trifascicular block	5 (7.7%)	2 (16.7%)	3 (5.7%)	
Pacing rhythm	6 (9.2%)	0 (0.0%)	6 (11.3%)	
IVCD	1 (1.5%)	0 (0.0%)	1 (1.9%)	
*Electrophysiological characteristics*				
His potential observed	23 (35.9%)	7 (58.3%)	16 (30.8%)	0.144
LBB potential observed	37 (56.9%)	10 (83.3%)	27 (50.9%)	0.085
*Pacing parameters*				
Sensed R wave amplitude (mV)	10.6 ± 5.0	10.7 ± 5.5	10.6 ± 4.9	0.970
Ventricular capture threshold (V)	0.9 ± 1.1	0.9 ± 0.3	1.0 ± 1.2	0.576
Ventricular pacing impedance (Ω)	675.1 ± 100.6	611.1 ± 100.3	689.5 ± 95.7	0.013
*QRS duration*				
Baseline QRS duration (ms)	128.6 ± 30.2	122.2 ± 32.8	130.0 ± 29.8	0.422
Post-LBBAP QRS duration (ms)	120.5 ± 20.1	114.7 ± 18.7	121.8 ± 20.3	0.269

Values are presented as mean ± standard deviation or *n* (%). LBBAP, left bundle branch area pacing; SSS, sick sinus syndrome; AV block, atrioventricular block; RBBB, right bundle branch block; LBBB, left bundle branch block; IVCD; interventricular conduction delay, LBB, left bundle branch; RV, right ventricle; LVAT, left ventricular activation time; ms, millisecond; min, minutes; mV, millivolt; Ω, ohm; *A*, atrial; *V*, ventricular.

**Table 3:** Procedural outcomes of LBBAP before and after reaching the learning curve.

	All (*N* = 50)	Early (1–24^th^ case)	Late (25–50^th^ case)	*P* value
*Acute outcome*				
Success of LBBAP	50/50 (100.0%)	24/24 (100.0%)	26/26 (100.0%)	1.000
Success of LBBP	49/50 (98.0%)	23/24 (95.8%)	26/26 (100.0%)	0.968
*Procedural characteristics*				
LBB potential	31 (62.0%)	13 (54.2%)	18 (69.2%)	0.451
Stim-LVAT (ms)	73.0 ± 10.7	73.1 ± 14.0	73.0 ± 6.8	0.959
Transition from nonselective LBB capture to selective LBB capture	6 (12.0%)	3 (12.5%)	3 (11.5%)	1.000
Numbers of attempts (*n*)	2.0 ± 0.9	1.8 ± 0.8	2.1 ± 1.1	0.604
Numbers of sheath change (*n*)	0.2 ± 0.5	0.2 ± 0.6	0.1 ± 0.3	0.319
Fluoroscopy time (min)	15.1 ± 13.5	20.5 ± 17.7	10.0 ± 3.6	0.009
Procedure time (min)	59.9 ± 24.8	68.1 ± 32.4	52.4 ± 10.9	0.032
*Pacing parameters*				
Sensed R wave amplitude (mV)	10.8 ± 4.8	11.8 ± 4.9	9.9 ± 4.6	0.186
Ventricular capture threshold (V)	1.0 ± 1.2	0.8 ± 0.3	1.2 ± 1.6	0.221
Ventricular pacing impedance (Ω)	675.7 ± 97.5	689.5 ± 84.3	663.0 ± 108.3	0.340
*QRS duration*				
Baseline QRS duration (ms)	127.7 ± 29.6	135.2 ± 29.5	120.8 ± 28.6	0.086
Post-LBBAP QRS duration (ms)	117.2 ± 19.0	113.2 ± 20.9	120.8 ± 16.6	0.156
*Complications*				
Complication	4 (8.0%)	2 (8.3%)	2 (7.7%)	1.000
Major complication	2 (4.0%)	2 (8.3%)	0 (0.0%)	0.435

Values are presented as mean ± standard deviation or *n* (%). LBBAP, left bundle branch area pacing; LBBP, left bundle branch pacing; LBB, left bundle branch pacing; stim-LVAT, stimulus to left ventricular activation time.

## Data Availability

The data underlying this article will be shared on reasonable request to the corresponding author.

## Abbreviations

AV: Atrioventricular
CSP: Conduction system pacing
DSP: Deep septal pacing
ECG: Electrocardiogram
HBP: His bundle pacing
LBB: Left bundle branch
LBBP: Left bundle branch pacing
LBBAP: Left bundle branch area pacing
LLL: Lumen-less pacing lead
LV: Left ventricular
RAO: Right anterior oblique
RBBB: Right bundle branch block
RV: Right ventricular
RVP: Right ventricular pacing
SDL: Stylet-driven pacing lead
SSS: Sick sinus syndrome
Stim-LVAT: Stimulus to left ventricular activation time.

## Supplementary Materials

Supplementary Materials Supplementary Figure S1: methods to find the optimal site for LBBAP. The His area tagging method (A) and the simplified nine-partition method (B) were presented. LBBAP, left bundle branch area pacing; LBBP, left bundle branch pacing; and RAO, right anterior oblique. Supplementary Figure S2: examples of intracardiac electrograms during the LBBAP. Stim-LVAT as measured in *V*5-*V*6 <75–85 ms and after further advancement of the lead tip, a transition from nonselective LBB capture (at 3.0 V) to selective LBB capture (at 2.0 V) is observed (A). The LBB potential is shown (B). LBBAP, left bundle branch area pacing; Stim-LVAT, stimulus to left ventricular activation time; and LBB, left bundle branch. Supplementary Figure S3: slope of the time required for LBBAP. The procedure time was modeled as cubic spline functions. The slope was calculated by differentiating the cubic spline curve with respect to the procedure time and the point where the slope becomes zero was the 24th case. LBBAP, left bundle branch area pacing.
