# Supplementary material for: Learning Curve Analyses for Left Bundle Branch Area Pacing with Conventional Stylet-Driven Pacing Leads
Source: J Interv Cardiol. 2023 May 18;2023:3632257. doi: 10.1155/2023/3632257 (PMC10212686; doi:10.1155/2023/3632257)
Supplement: Supplementary Materials — Supplementary Figure S1: methods to find the optimal site for LBBAP. The His area tagging method (A) and the simplified nine-partition method (B) were presented. LBBAP, left bundle branch area pacing; LBBP, left bundle branch pacing; and RAO, right anterior oblique. Supplementary Figure S2: examples of intracardiac electrograms during the LBBAP. Stim-LVAT as measured in V5-V6 <75–85 ms and after further advancement of the lead tip, a transition from nonselective LBB capture (at 3.0 V) to selective LBB capture (at 2.0 V) is observed (A). The LBB potential is shown (B). LBBAP, left bundle branch area pacing; Stim-LVAT, stimulus to left ventricular activation time; and LBB, left bundle branch. Supplementary Figure S3: slope of the time required for LBBAP. The procedure time was modeled as cubic spline functions. The slope was calculated by differentiating the cubic spline curve with respect to the procedure time and the point where the slope becomes zero was the 24th case. LBBAP, left bundle branch area pacing. [file 3632257.f1.pdf]

Supplementary Figure S1. Methods to find the optimal site for LBBAP.

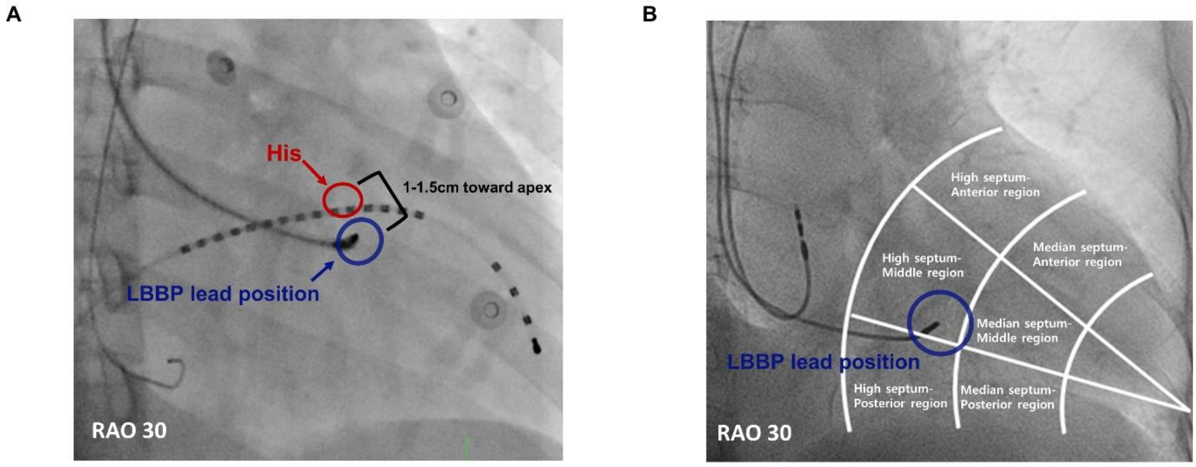

The His area tagging method (A) and the simplified nine-partition method (B) were presented.

LBBAP, left bundle branch area pacing; LBBP, left bundle branch pacing; RAO, right anterior oblique.

Supplementary Figure S2. Examples of intracardiac electrograms during the LBBAP.

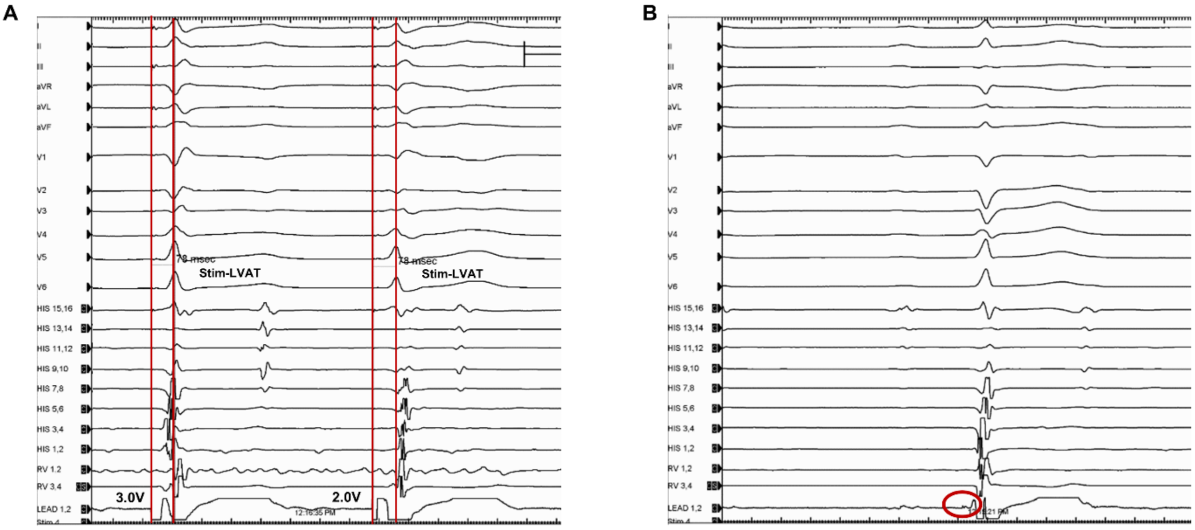

Stim-LVAT as measured in V5-V6 <75–85 ms and after further advancement of the lead tip, a transition from nonselective LBB capture (at 3.0V) to selective LBB capture (at 2.0V) is observed (A). The LBB potential is shown (B).

LBBAP, left bundle branch area pacing; Stim-LVAT, stimulus to left ventricular activation time; LBB, left bundle branch.

Supplementary Figure S3. Slope of the time required for LBBAP.

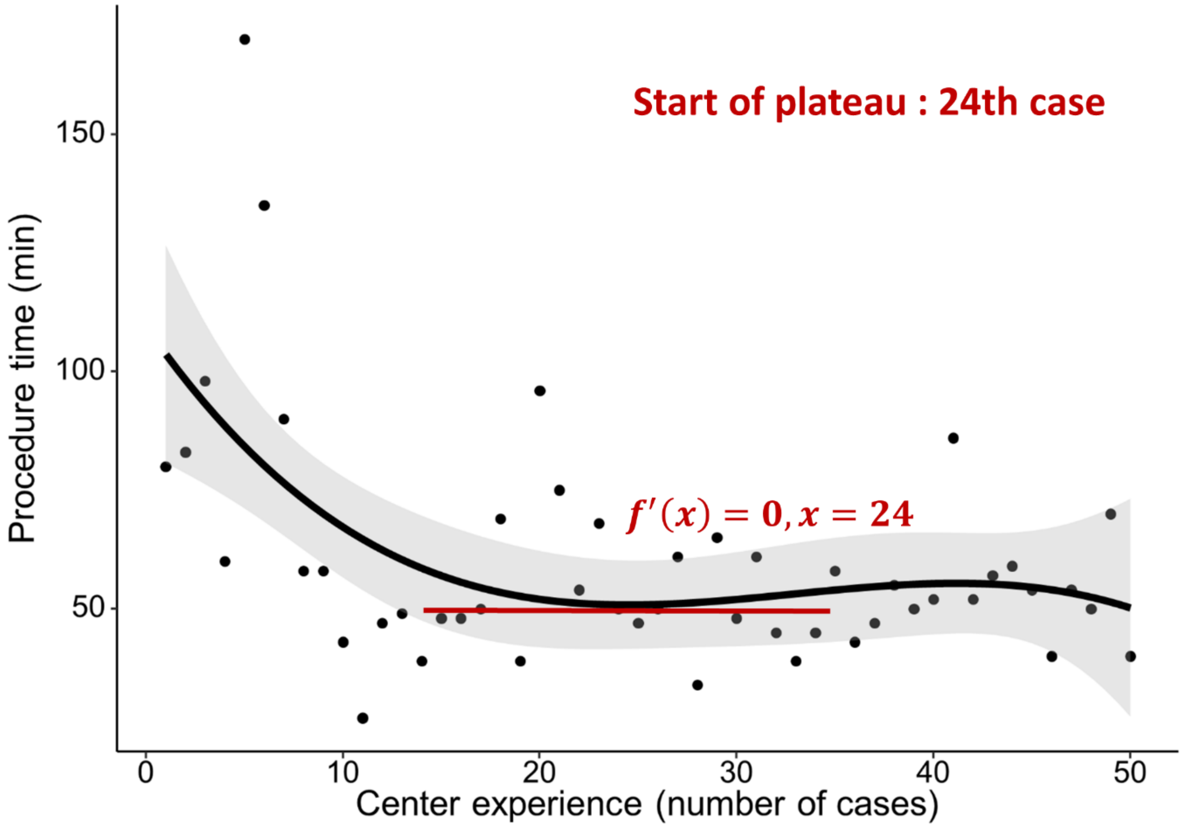

The procedure time was modeled as cubic spline functions. The slope was calculated by differentiating the cubic spline curve with respect to the procedure time and the point where the slope becomes zero was the 24th case.

LBBAP, left bundle branch area pacing.
